# Candidate genes associated with bud dormancy release in blackcurrant (*Ribes nigrum *L.)

**DOI:** 10.1186/1471-2229-10-202

**Published:** 2010-09-14

**Authors:** Peter E Hedley, Joanne R Russell, Linzi Jorgensen, Sandra Gordon, Jenny A Morris, Christine A Hackett, Linda Cardle, Rex Brennan

**Affiliations:** 1 Programme of Genetics, SCRI, Invergowrie, Dundee, UK; 2 BioSS, Invergowrie, Dundee, UK

## Abstract

**Background:**

The detrimental effects of mild winter temperatures on the consistency of cropping of blackcurrant (*Ribes nigrum *L.) in parts of Europe have led to increasing interest in the genetic control of dormancy release in this species. This study examined patterns of gene expression in leaf buds of blackcurrant to identify key differential changes in these profiles around the time of budbreak.

**Results:**

Using leaf bud tissue of blackcurrant, a cDNA library was generated as a source of blackcurrant ESTs for construction of a custom microarray, which was used to identify differential gene expression during dormancy release. Gene activity was lowest in early stages of dormancy, increasing to reach a maximum around the time of budbreak. Genes with significantly changing expression profiles were clustered and evidence is provided for the transient activity of genes previously associated with dormancy processes in other species. Expression profiling identified candidate genes which were mapped onto a blackcurrant genetic linkage map containing budbreak-related QTL. Three genes, which putatively encode calmodulin-binding protein, beta tubulin and acetyl CoA carboxylase respectively, were found to co-localise with budbreak QTL.

**Conclusions:**

This study provides insight into the genetic control of dormancy transition in blackcurrant, identifying key changes in gene expression around budbreak. Genetic mapping of ESTs enabled the identification of genes which co-localise with previously-characterised blackcurrant QTL, and it is concluded that these genes have probable roles in release of dormancy and can therefore provide a basis for the development of genetic markers for future breeding deployment.

## Background

Blackcurrant (*Ribes nigrum *L.) is grown widely throughout temperate areas of Europe and New Zealand, with an annual production of 160,000 tonnes in Europe and 185,000 tonnes globally [1]. The centres of genetic diversity for blackcurrant are situated in northern Scandinavia and Russia, although species are also found in North and South America, Asia and northwest Africa [2]. The fruit has very high nutritional value, in terms of the content of ascorbic acid and other antioxidants [3], and its primary commercial use is processing for juice. In the UK, varieties are chosen to give a spread of cropping seasons. Timing of leaf budbreak is an important physiological factor in the developmental processes leading to cropping of blackcurrant, as uniformity of development is essential for both successful harvesting and for optimum fruit quality and yield.

The control of budbreak in many woody perennial plants, including berry fruits such as *Ribes *and *Rubus*, is dependent on exposure to chilling temperatures during the winter for a sufficient duration to release dormancy, followed by appropriate warmer temperatures in the spring to induce growth [4]. An insufficient amount of chilling during the dormant period can seriously impact key phenological traits, notably time of budbreak, time and duration of flowering and fruit quality at harvest [5]. The chilling requirement, in terms of both duration and range of suitable temperatures, varies between species, between cultivars within species [6,7], and even between different buds of the same plant [8,9]. Blackcurrant has a relatively high chilling requirement for dormancy break, from *ca*. 1,300 h in the case of New Zealand-selected germplasm [10] to over 2,000 h for some late-flowering types such as 'Ben Lomond' from Scotland and most Nordic cultivars [7]. As warmer winters are likely to become increasingly prevalent [11], there is a risk of insufficient chilling becoming more widespread in cultivars requiring high levels of winter chilling, so breeding strategies to address this using low-chill parental material are now required [7].

However, these must be balanced against the risk of spring frost damage at flowering in cultivars that break bud too early after minimal chilling and whilst the incidence of spring frosts is declining across Europe [5], there can still be occasional damage in some areas. Also, the risk of spring frost damage at flowering may depend on temperature variance as well as on specific mean temperatures, and climate projections are predicting a future rise in such variance [12].

Dormancy in woody perennials is an adaptive mechanism for the survival of winter conditions [9] and was classified by Lang [13] into three major types: (i) ecodormancy, where growth inactivity is caused by unfavourable environmental conditions, with a resumption of growth when conditions improve; (ii) endodormancy, caused by endogenous factors within the dormant buds that cannot be overcome even by favourable environmental conditions; and (iii) paradormancy, or correlative inhibition [14], where the dormancy status of buds is influenced by physiological factors in other parts of the plant (eg. apical dominance). The effects of winter chilling on dormancy break at bud-burst are in endodormant tissues, and further investigation of winter chilling in blackcurrant is structured around improved phenotyping of diverse germplasm [7] coupled with genetic analysis of Quantitative Trait Loci (QTL) and genes associated with dormancy-related processes. Putative QTL linked to key developmental traits, including time of budbreak, have recently been identified by Brennan *et al. *[15].

Dormancy break and subsequent physiological events are controlled through the coordinated action of large numbers of genes in woody plants [9] and whilst there have been many studies of the molecular genetics of dormancy and budbreak, many aspects remain unclear. Microarray studies to examine global gene expression during dormancy and budbreak have been used in a range of woody genera, including *Vitis*, [16-18], *Quercus *[19] and *Rubus *[20]. Most of these studies attempted functional classification of the differentially expressed genes from the transcriptome related to budbreak or, in the case of Mathiason *et al. *[18], to the fulfilment of chilling requirement. Some studies, eg. Mazzitelli *et al. *[20] and Derory *et al. *[19], have identified candidate genes for dormancy-related metabolism, and several of these genes were previously implicated in these processes in other species.

The aim of this study was to examine patterns of gene expression in leaf buds of blackcurrant and to identify the key differential changes in these profiles around the time of budbreak. A custom cDNA microarray was utilised which incorporated blackcurrant Expressed Sequence Tags (ESTs) derived from buds at various stages of dormancy. Expression profiling identified candidate genes associated with budbreak which were mapped onto a blackcurrant genetic linkage map. Initial steps have been taken to develop associated markers that can be deployed in the characterisation of diverse germplasm for inclusion in downstream breeding programmes.

## Results

### Blackcurrant expressed sequence tags

RNA was isolated from blackcurrant leaf bud tissue sampled at five time-points prior to budbreak and, following pooling, was used to construct a targeted cDNA library to maximise collation of relevant ESTs. Single-pass sequencing of approximately 9,000 clones generated 7,400 high-quality ESTs. Following identification of a low-redundancy set of blackcurrant clones, single ESTs (3,633 in total) representing each contiguous sequence were selected and whole cDNA inserts used to fabricate microarrays as described.

### Gene expression profiling during dormancy transition

Leaf buds were sampled from blackcurrant plants throughout the different dormancy phases, from mid-winter following onset of dormancy through to spring when bud-burst was prevalent. RNA was isolated from bud samples from winter 2005 to spring 2006: week 0 (early December), week 2, week 4, week 8, week 12, week 14 and week 16 (end of March). For the microarray experimental design, RNAs from consecutive time-points were hybridised on the same arrays in a two-channel loop-design (design and complete dataset available at ArrayExpress [21] accession E-TABM-807). Data were extracted from each array and quality filtering was applied to leave 2,320 reliable replicated expressed probes.

**Figure 1:**
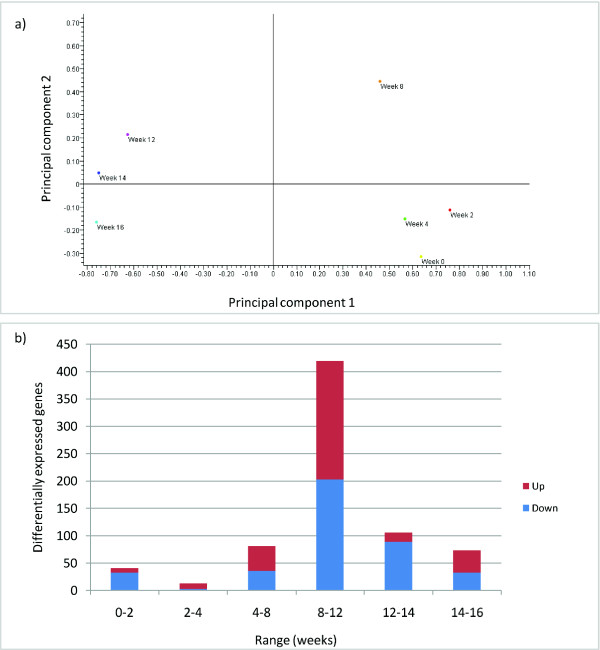
**Overview of *Ribes *gene expression profiling**. a) Principal component analysis (PCA) of microarray expression data based upon sample time; b) Frequency of significant differentially expressed genes between sample time points. Scale: x-axis, range in time of sampling (weeks); y-axis, number of differentially expressed genes. Red: up-regulated; blue: down-regulated.

In order to identify and characterise variation within the entire reliable dataset Principal Component Analysis (PCA) was used (Figure 1a). Principal component 1 accounted for 66% of the variance, whereas principal component 2 comprised 12% of the variance, based upon the filtered genelist of 2,320 probes. Principal component 1 separates the samples of the earlier weeks (0, 2, 4 and 8) from those of the later weeks (12, 14 and 16). Principal component 2 in particular separates the week 8 data from the other time points. To analyse this further, an overview of gene activity in blackcurrant leaf bud tissue was obtained using volcano plots to identify numbers of differentially expressed genes between consecutive sample time points (Figure 1b). Early winter (week 2 to week 4) shows the lowest levels of differential gene expression, and in the first two weeks of sampling the majority of genes are down-regulated. Differential gene expression reaches a peak between week 8 and week 12, with similar numbers of up-and down-regulated genes detected. Subsequently, the majority of genes from week 12 to week 14 are reduced in expression levels. 

**Figure 2:**
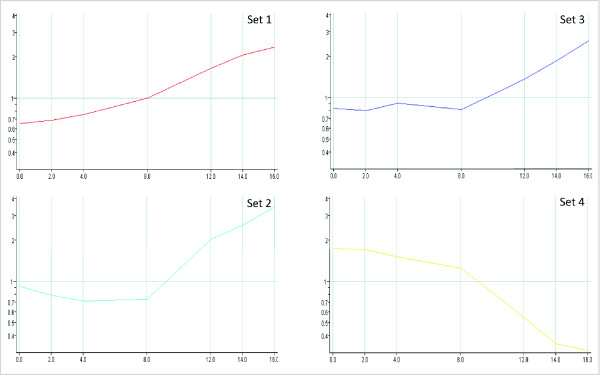
**Clustering of *Ribes *gene sets**. K-means clustering of significant gene expression profiles into 4 gene sets. Scale: x-axis, time of sampling (weeks); y-axis, fold-change (log scale). Average profiles are shown.

Significant differentially expressed genes were identified over the entire period of sampling using ANalysis Of VAriance (ANOVA). False Discovery Rate (FDR) was applied at high stringency multiple testing correction; identifying 1,040 significantly changing probes (Additional File 1), of which 842 unigenes have significant homology to known *Arabidopsis *gene loci. The high proportion of significantly changing genes reflects our generation and use of a microarray targeted to dormancy processes in *Ribes*. Unigenes considered to have significantly changing gene expression profiles following ANOVA analysis were subjected to K-means clustering to group similarly regulated genes together (Figure 2). For simplicity, average gene expression profiles are shown for each group to indicate general trends. The four groups obtained (Set 1-Set 4, Figure 2) consist of 260 (227 annotated), 181 (160), 124 (107) and 475 (348) unigenes respectively. 

To obtain a general overview of putative gene function within significant gene clusters, individual gene sets were analysed for enrichment of Gene Ontology (GO) terms using the Term Enrichment Tool at AmiGO [22]. Frequencies of GO categories within the blackcurrant homologue lists were compared to those found in the whole microarray set and these are represented in Additional File 2. All four groups showed significant enrichment of GO terms, thereby linking putative function with temporal gene expression patterns: Set 1 shows a gradual and consistent increase from week 0 to week 8, followed by more rapid induction to week 16, and the majority of overrepresented terms in this group appear to be associated with protein biosynthesis and metabolism. Set 2 does not show induction until week 8, increasing rapidly through the remaining sample points, and this group also shows enrichment for general cellular metabolism and biosynthesis, but also contains terms related to photosynthesis. Set 3 shows initial reduction in expression from week 0 to week 4 and is subsequently induced dramatically from week 8, and the majority of terms overrepresented in this group are associated with secondary metabolism. Set 4 shows gradual down-regulation from week 0 to week 8, followed by rapid decline through to week 16, and this set is enriched for transcription factor (TF) terms. Individual genes and their homologies will be discussed in the context of specific metabolic processes and putative biological function later in this manuscript.

### Validation of microarray gene expression profiling

**Figure 3:**
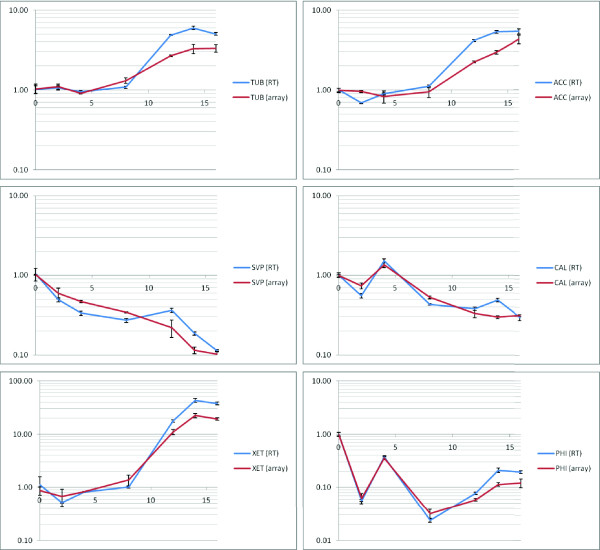
**Validation of selected *Ribes *gene expression profiles**. Validation of microarray gene expression data using real-time RT-PCR assays: TUB, beta-2 tubulin (GT026930); ACC, acetyl-CoA carboxylase (GT022654); SVP, MADS-box protein (GT022063); CAL, calmodulin-binding protein (GT027101); XET, xyloglucan endotransglucosylase (GT026044); PHI, phi-1 (GT025772). Scale: x-axis, time of sampling (weeks); y-axis, fold-change (log scale). Bars represent standard error.

In order to validate gene expression profiles obtained from the microarray data, a subset of eighteen genes were identified from the significant ANOVA gene-list with contrasting patterns and magnitude of regulation. Sequences from the associated ESTs were used to design real-time RT-PCR assays (Additional File 3). Comparison of gene expression profiles between microarray data and real-time RT-PCR data is presented in Figure 3 and Additional File 4, indicating clear correspondence between the two independent expression technologies in the context of this study.

### Genetic mapping and association of candidate genes with dormancy traits

**Figure 4:**
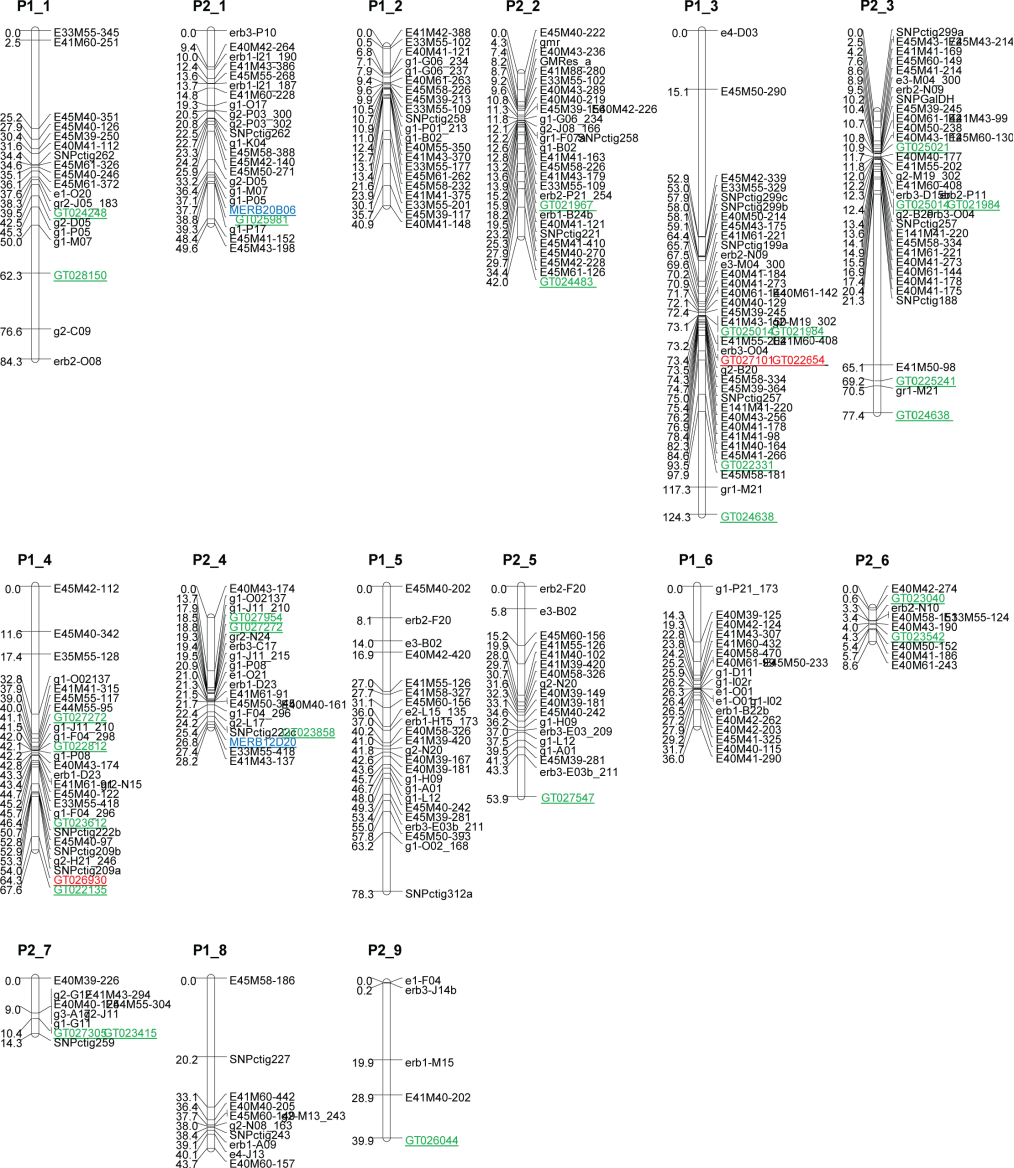
**Linkage map of *Ribes***. Linkage map for the two parents of the mapping population; P1 is the seed parent SCRI 36/1/100 and P2 is the pollen parent S10-2-27/28. Markers derived from the microarray analysis are coloured: red, candidate genes associated with significant budbreak QTL; green, genes mapped by SNP assays; blue, genes mapped by SSR assays.

In order to map candidate genes, single nucleotide polymorphisms (SNPs) or simple sequence repeats (SSRs) were identified from the candidate genes using genomic DNA from parents and progeny of a well characterised mapping population (S36-1-100 × S10-2-27/28) which segregates for several QTL, some of which are directly related to budbreak and dormancy characters [15]. A subset of ESTs was selected from the significant gene-lists for full-insert sequencing (Additional File 5). Selection was based upon levels of significance with respect to differential gene expression and ability to define putative annotation from gene homologues. Primers were designed to original EST sequences for 112 (48 up-regulated and 64 down-regulated) genes. Single PCR products were successfully amplified from 89 of these, of which 47 sequences were polymorphic, with at least one SNP per sequence. Pyrosequencing assays were designed to 41 of these SNPs for subsequent mapping in the complete blackcurrant population. From these, 24 SNP-markers segregated in the expected ratios (either 1:1 (homozygous: heterozygous) if just one parent was heterozygous, or 1:2:1 (hom:het:hom) if both parents were heterozygous) among the progeny and were mapped to the linkage map (Figure 4). As well as SNPs, we also identified SSRs for which primers could be designed to 16 of the 112 genes. These were tested on the mapping population and, of the three which generated polymorphic loci, two were mapped to genetic locations on the linkage map (Figure 4).

Quantitative trait loci interval mapping previously identified a QTL for budbreak in 2002 on linkage group 3 [15], a region which also contains SERB09L03 (GenBank accession GT022654) and SERB03C05 (GT027101). The recombination frequency between these SNP-markers was zero. The maximum LOD (logarithm (base 10) of odds) score in this region was 3.4. In 2003, the LOD score for budbreak in this region was less than the usual threshold of 3.0, but the QTL effect was the same sign [15]. A more powerful QTL by environment (year) analysis of the combined 2003 and 2004 data [23] showed that SERB09L03 (GT022654) and SERB03C05 (GT027101) were significantly associated with budbreak (p-value < 0.001) and that there was no significant QTL by year interaction. The mean budbreak for the heterozygous genotype at these SNPs was 4.0 days (se 2.35) earlier than the homozygote in 2002 and 1.6 (se 0.84) earlier in 2003. These two ESTs encode proteins with homology to an acetyl-CoA carboxylase (blastx 2e-47) and a calmodulin-binding protein (blastx 2e-17), respectively. Another EST, SERB02K12 (GT026930) on linkage group 4, was found using the QTL by environment analysis to be associated with budbreak (p-value < 0.001). The mean budbreak for the heterozygous genotype at this SNP was 2.5 days (se 1.38) later than the homozygote in 2002 and 2.1 (se 1.44) later in 2003. This has derived amino acid homology to a beta-tubulin (blastx 1e-94). Specific gene expression profiles, including quantitative RT-PCR validation, is presented in Figure 3 and indicates that the genes encoding acetyl-CoA carboxylase and beta-tubulin have similar patterns, with relatively constant expression to week 8, followed by rapid accumulation of transcripts later in the time course. The gene encoding the calmodulin-binding protein however, initially dips in activity at week 2, recovers and gradually declines through the remainder of the sampling period.

## Discussion

The need for blackcurrant varieties requiring lower levels of chilling during the dormant period is likely to increase in the future [7], although current unpredictability of weather events in many of the growing regions means that utilising a range of varieties with varying chilling requirements is probably advisable for most growers. For example, varieties that break bud too early may be vulnerable to damage by spring frosts during the flowering period in some northern areas, notably Scandinavia [24]. However, the selection of varieties based on budbreak characteristics currently depends largely on phenotypic assessments in the field using prevailing weather conditions. To move beyond this into a more focused selection procedure requires detailed knowledge about the genetic control of budbreak and subsequent developmental events in blackcurrant, which will enable development of molecular markers directly linked to traits of interest.

### General observations of gene activity in blackcurrant bud tissue

This study provides insight into the genetic components associated with dormancy transition in blackcurrant. A targeted blackcurrant cDNA microarray was constructed and utilised to identify sets of genes with significant differential expression profiles during dormancy release and budbreak. Utilising PCA and identifying differentially expressed probes between sampling stages clearly indicated maximum gene activity between week 8 and week 12 (Figure 1), during the month of February. This was also demonstrated in all four of the primary gene expression profiles determined by K-means clustering (Figure 2), with rapid induction or repression after week 8. Up to this point, the cumulative chilling (temperatures below 7°C) was 1,723 h (see Methods), similar levels to which have been demonstrated under controlled environments in *Rubus *to develop 50-100% budburst in whole canes or isolated nodes respectively [20]. This was slightly lower than that estimated by Fraser [25] using cuttings, rather than whole plants, which indicated the chilling requirements of commercial UK cultivars of blackcurrant, including 'Ben Gairn' and 'Ben Hope', to be > 2000 h. In *Vitis riparia*, almost 100% budburst was achieved in canes after 1,500 h of chilling [18]. Accumulated hours of chilling in our study would indicate that our material received sufficient chilling for budbreak between week 8 and week 12 and, combined with the general observations in gene expression profiles, suggest that the blackcurrant samples include bud material in transition from the endodormant to ecodormant phases [25].

Putative roles of the significantly changing genes during dormancy release from this study remain speculative and care must be taken in interpreting the data, however a number of gene homologues identified have been previously described in several related studies. Some of these common genes of interest are briefly discussed in the context of dormancy transition and budbreak.

### Association of dormancy transition with signalling, transcription factors and secondary metabolism

Almost half of the significant differentially expressed blackcurrant genes clustered into expression profile Set 4 (Figure 2), whereby gene activity in general was highest in early winter, subsequently declining throughout the remaining sampling period into spring-time. This set was overrepresented by GO terms associated with transcription factors (TFs; Additional File 1). Of note within this group are genes encoding TFs (see Additional File 4), of which there are several zinc finger family members (B-box, CCCH, C2H2, and C3HC4/RING types) which are known to play important roles in signal transduction pathways of plants [26]. A zinc finger protein from *Quercus *[19] has been implicated in budburst, which is related to a TF involved in seed germination from *Arabidopsis *[27]. Another class of TF which shows gradual down-regulation through the time course in *Ribes *encodes a Short Vegetative Phase (SVP) type MADS box transcription factor (Additional File 4). Homologues of SVP have been identified in Japanese apricot [28] and raspberry [20], where the gene has similar expression patterns to that observed in blackcurrant under controlled conditions leading to dormancy release. SVP has been shown in *Arabidopsis *to act as a direct repressor of flowering [29] and appears to have elevated expression under short-day environments [30]. A TF family highly related to SVP, termed Dormancy Associated MADS-box (DAM), has been described in several other perennial species, including poplar [31], leafy spurge [32,30] and peach [33]. Expression of two leafy spurge genes encoding DAM proteins reflect the dormancy status within the leaf meristem, with both genes induced by cold temperatures and differentially expressed according to day length. MADS-box TFs are clearly important components in signalling and dormancy transition in several species.

Oxidative stress has been reported to be an important factor in controlling budbreak, and induction of dormancy release by artificial stimuli, such as heat stress and hydrogen cyanamide, leads to the differential expression of several genes associated with this process in grape [34]. From the blackcurrant array data, there is evidence of transient expression of several genes whose products are coupled with this mechanism (Additional File 4), including ascorbate peroxidase (Set 1), calcium-dependent protein kinases (Set 1 & Set 4), glutathione S-transferases (GST; Set 1 & Set 4), catalase (Set 4) and sucrose synthase (Set 4). Similar mechanisms appear to also be involved in dormancy release of raspberry leaf buds, with transient induction of genes encoding ascorbate peroxidase, catalase and GST [20]. Other signalling systems, demonstrated by artificial dormancy control in grape, involve ethylene and abscisic acid (ABA), leading to the hypothesis that there is complex interaction between these mechanisms to control dormancy release [35]. Within the blackcurrant data, there is evidence of ethylene signalling during transition with transient expression of several ethylene response factors (ERFs; Set 4), which have also been observed in leafy spurge [36]. Many of the blackcurrant genes strongly induced later in the time-course (Set 3, Figure 2) are associated with secondary metabolism, specifically those leading to flavonoid biosynthesis. These include genes encoding chalcone isomerase, chalcone synthase, dihydroflavonol reductase and flavonol synthase (Additional File 4), some of which have also been reported to be significantly regulated during dormancy release in raspberry [20] and leafy spurge [14,37]. Flavonoids provide protection of tissues against UV damage (reviewed in [38]) and are known to control auxin transport in plants, subsequently altering growth of tissues such as axillary buds [39].

### Candidate genes associated with dormancy traits

Genetic mapping of significantly expressed ESTs enabled identification of three genes which co-localise with previously characterised blackcurrant budbreak QTL [15], two of which map with high significance to a single locus on linkage group 3 and another which maps to linkage group 4 (Figure 4). These associations provide independent evidence that these genes may be implicated in dormancy processes, however it should be noted that they may also be simply co-located with the causal genes. Each of these ESTs is discussed on the basis of their homologies to known genes and gene products, and in the context of previous work on dormancy in other species. 

Acetyl CoA carboxylase (ACCase, encoded by GT022654) functions to provide malonyl-CoA as a substrate for biosynthesis of fatty acids [40] and secondary metabolites, such as flavonoids and anthocyanins [41] which are essential for plant development and protection. It is known that the homomeric form is induced by UV-B radiation for flavonoid production [42]. The gene expression profile derived from this EST (Figure 3) is a member of Set 3 of the clustered groups (Figure 2), which has overrepresentation of terms associated with secondary metabolism and phenylpropanoid biosynthesis. ACCase is associated with rapidly dividing tissues and early stages of cell growth and development and this gene is clearly induced in expression during early spring. In addition, an ACCase has been shown to be induced in floral buds of common bean [42].

Clone GT027101 encodes a gene product with homology to calmodulin-binding protein. Calmodulin itself binds to calcium, which is common regulator of many different protein targets and a transducer of secondary messenger signals [43]. As previously mentioned, oxidative stress is also known to induce calcium signalling in plant cells [34]. Calmodulin-binding proteins exist in many different forms with a range of associated functions and have been demonstrated to control calcium signalling in woody perennial species such as grape [44] and blueberry [45]. The expression profile of the gene in blackcurrant (Figure 3) indicates repression from week 4 and is present within cluster Set 4 (Figure 2), which contains other genes with GO terms associated with transcription factors. The polypeptide encoded by this gene also shows similarity to a putative pheromone receptor protein AR781 in *Arabidopsis*, whose EST profiles show high levels of expression in bud and seed tissue (data from dbEST [46]; Id 5922760).

A third blackcurrant gene (GT026930), which maps to a budbreak QTL on linkage group 4, encodes a protein with high homology to beta-tubulin, which is an essential component of microtubules within the cytoskeleton. High levels of beta-tubulin expression is a clear indicator of rapidly dividing and expanding cells and the gene expression profile shows rapid induction after week 8 (Figure 2). This profile is a member of Set 1 (Figure 3) which contains many terms representative of increased protein metabolism and biosynthesis. From previous studies, beta-tubulin is consistently induced on dormancy release in buds of a range of woody perennial species, including *Malus *and *Rosa *[47], and is proposed as a genetic marker for dormancy status in such species. It has also been demonstrated that a beta-tubulin gene is induced in expression during bud-burst in poplar [48].

Extending this study to look for association of these genes with dormancy-related traits in a wide blackcurrant germplasm collection is planned and will further substantiate their roles in physiological processes related to budbreak. However, it is clear that they have excellent potential as genetic markers to assist future breeding strategies. Further work to confirm the genetic map locations for both phenological and fruit quality traits is currently in progress with an extended blackcurrant mapping population and appropriate phenotyping.

## Conclusions

This study provides insight into the genetic control of dormancy transition in blackcurrant, and the use of microarrays over a time course leading up to budbreak shows key changes in gene expression at budbreak. Further mapping of ESTs with significant differential changes in expression around budbreak enabled the identification of three genes which co-localise with previously-characterised blackcurrant QTL, and it is concluded that these genes have probable roles in the dormancy and budbreak processes, and can therefore provide a basis for the development of genetic markers for future breeding deployment.

## Methods

### Plant material

Blackcurrant (*Ribes nigrum *L. cv. Ben Hope) plants were grown in the field at SCRI, Dundee, Scotland (56° 27'N, 3° 04'W) to four years of age. Leaf buds were sampled at eight time-points: week 0, 7/12/05; week 2, 21/12/05; week 4, 04/01/06; week 8, 01/02/06; week 12, 01/03/06; week 14, 16/03/06; week 16, 29/03/06; week 43, 16/10/06. Air temperature measurements were taken from October and cumulative hours of chilling (below 4°C) determined: week 0, 610 h; week 2, 837 h; week 4, 1,140 h; week 8, 1,723 h; week 12, 2,262 h; week 14, 2,622 h; week 16, 2,847 h. Buds were taken from three biological replicate pools at random positions within the bush, flash frozen in liquid nitrogen and stored at -80°C. Candidate genes were mapped using the reference blackcurrant population designated 9328, described by Brennan *et al *[15].

### RNA extraction

Total RNA was extracted from frozen blackcurrant bud material (100 mg) using the Plant RNeasy Mini Extraction Kit (Qiagen) according to the manufacturer's recommendations (substituting buffer RLC for RLT and including 10% v/v RNA Isolation Aid (Ambion) and 1% v/v β-mercaptoethanol). RNA quality was checked by spectrophotometry and integrity assessed using a Bioanalyzer (Agilent Technologies).

### cDNA library construction and EST sequencing

Only very limited numbers of publicly accessible gene sequences are available for blackcurrant (less than 30 sequences present in NCBI [49], 2009) and closely related woody perennial species. Therefore a cDNA library was generated from blackcurrant leaf bud tissue, which was used as a source of probes for construction of a custom microarray. Utilisation of these expressed gene sequences maximised the chances of identifying putative candidate genes associated with dormancy traits and budbreak in blackcurrant. Total RNA from blackcurrant buds (equal quantities of each) were pooled from five time-points (week 0, week 2, week 4, week 8 and week 12) prior to and potentially including budbreak. Messenger RNA was purified from 100 μg total RNA using Dynabeads mRNA Purification Kit (Invitrogen) as recommended. A directional cDNA library was constructed in pSPORT1 from pooled mRNA using the Superscript Plasmid System (Invitrogen) according to the manufacturer's instructions. Randomly selected clones (~9,000) were picked and stored as glycerol stocks. From these, plasmids were prepared using Multiscreen 96-well Filter Plates (Millipore) as recommended. Sequencing reactions were carried out using M13 reverse primer and BigDye version 3.1 Kit (Applied Biosystems) according to the manufacturer's instructions. Completed sequencing reactions were purified using ethanol precipitation prior to analysis on an ABI PRISM 3730 DNA Sequencer (Applied Biosystems). Sequences were trimmed according to chromatogram quality criteria, using the base-calling program Phred [50] at a score >20, and vector-derived sequences were removed using Crossmatch software [50].

In order to estimate the redundancy of the ESTs, related cDNA sequences were assembled as contiguous sequences using the CAP3 program [51] with default settings of all parameters [52]. ESTs were defined as redundant when they exhibited more than 95% identity over aligned regions and were assembled into a single contiguous sequence.

Similarities to previously identified sequences were obtained by searching public databases using the BLASTn and BLASTx algorithms [53] for nucleotide and deduced amino acid sequences respectively. Local databases containing non-redundant nucleotide and protein sequences obtained from the National Center for Biotechnology Information [21] were searched. Matches were considered non-significant (no match) when e-values were greater than 0.01. All EST sequences used to fabricate microarrays have been submitted to dbEST at GenBank [46].

### Microarray fabrication

Blackcurrant cDNA inserts were amplified by PCR using plasmid DNA template and M13 forward and reverse primers that span the multiple cloning site of the vector. Each reaction was performed in 100 μl containing 50 ng of plasmid, M13 primers (0.5 μM each), 0.2 mM dNTPs, 2 mM dNTPs, 2 mM MgCl_2 _and 0.10 U/μl Taq DNA Polymerase (Promega) in 1 × PCR buffer. PCR conditions were 94°C for 3 min for 1 cycle and then 94°C for 30 s, 54°C for 30 s, 72°C for 2 min for 38 cycles, 72°C for 7 min for 1 cycle. PCR products were purified using the MinElute 96 UF PCR Purification Kit (Qiagen) following the manufacturer's recommendations. Purified PCR products were standardised to 400 μg/μl.

A total of 3,633 cDNA amplicons from blackcurrant library clones were prepared for spotting onto modified glass slides (Nexterion Slide A, Schott Glass). Purified amplified cDNA (7.5 μl at 400 μg/μl) was mixed with 2.5 μl of 99% dimethylsulfoxide (DMSO), and transferred to 384-well print plates (Genetix). Probes were printed in triplicate with a defined random pattern onto each slide using a 24-pin robotic system (Q-Array Mini, Genetix) at constant relative humidity (50%) at 23°C. Arrays were allowed to air-dry for 10 min and the cDNA spots were immobilised following the Nexterion protocol. The quality of the printing process was checked using the SpotCheck Microarray Slide QC kit (Genetix). Details of the blackcurrant dormancy array design can be found at ArrayExpress (accession A-MEXP-1694; [22])

### Microarray processing

Target total RNA (2 μg) was labelled using the 3DNA Array 900 Kit (Genisphere) as recommended. RNAs from consecutive time-points were hybridised on the same arrays in a two-colour loop-type design (see ArrayExpress accession E-TABM-807 [22]) incorporating three biological replicates for each time point (8 time points, 24 samples, 12 arrays total).

Arrays were scanned using an ArrayWoRx Auto scanner (Applied Precision) at appropriate exposure settings for Cy3 (595 nm) and Cy5 (685 nm) at 9.75 μm resolution, generating separate TIFF images. Exposure levels were adjusted to compensate for slight variations in labelling efficiencies. Data were acquired from images using GenePix Pro software (Molecular Devices) and median signal and background intensities were determined for the Cy3 and Cy5 channels for each spot on each microarray. Background-subtracted intensity values were imported into GeneSpring (v.7.3; Agilent Technologies), whereby data from replicate spots within each array were averaged.

### Microarray data analysis

Data sets for each array were normalised in GeneSpring using the LOWESS (LOcally WEighted polynomial regreSSion) algorithm to minimize differences in dye incorporation efficiency [54], prior to re-importing the data as single colour intensity values. Subsequent analysis was carried out using only the first seven sampling points (week 0, week 2, week 4, week 8, week 12, week 14 and week 16) representing winter-spring seasons. Scaling of the data was performed using default GeneSpring settings. Data were filtered by expression level (>100 in 3/21 samples) to remove unreliable, low intensity data points, leaving 2,320 genes for downstream analysis. Data from biological replicates were combined in GeneSpring for statistical analysis: (i) volcano plots (p-value <0.05, fold-change >2 ×) were used to identify differentially expressed genes between sample time points; (ii) 1-way ANOVA was used to estimate differential response between time points (default parameters: parametric test, variances not assumed equal (Welch ANOVA)) and probes were selected (1,040 genes) that were judged to be statistically significant (p-value <0.01) with Benjamini & Hochberg multiple testing correction [55].

Clustering of gene expression profiles across the developmental time series was performed in GeneSpring using the K-means algorithm. Default parameters (100 iterations, Pearson measure as similarity correlation) were used to generate 4 cluster sets from the selected ANOVA gene-list.

### Analysis of Gene Ontology terms

Blackcurrant unigenes used to construct the microarray were annotated with the most significant BLAST homologue to *Arabidopsis *gene loci. These homologues from selected gene-lists were subsequently analysed for enrichment of Gene Ontology (GO) terms using the Term Enrichment Tool [56] at AmiGO (version 1.7; [24]). The whole microarray gene set was used as the defined background and thresholds of p-value 0.05 and a minimum of 1 gene product were used.

### Real time RT-PCR validation

Contiguous EST sequences containing probes selected following microarray analysis were used to design Universal Probe Library (UPL, Roche) assays using default parameters [57]. Primer and probe sequences are detailed in Additional File 3 and were designed to the three candidate genes described and also fifteen additional randomly selected unigenes with contrasting expression patterns. Total RNA (5 μg) was reverse transcribed using oligo d(T)_18 _and You-Prime RT Beads (GE Healthcare) as recommended. cDNA was diluted to 50 μl and 2 μl of each sample used as template for UPL assays with FastStart TaqMan Probe Master ROX (Roche) as recommended, with standard cycling and data acquisition on 7500 Fast Start (Applied Biosystems) equipment. Assay efficiencies were evaluated with standard curves for reference (*eIF4A*) and each test gene, which determined if ΔΔCt or Pfaffl [58] calculations were used to estimate relative gene expression profiles.

### Candidate gene mapping

Inserts from 112 selected EST clones (Additional File 5) identified by ANOVA filtering were completely sequenced using Sanger technology as above, independently with both M13 forward and reverse priming. Sequences were assembled using Sequencher software (v. 4.9, Genecodes) with default parameters. Oligonucleotide primers (Additional File 5) were designed using the Primer 3 programme [59]. If the lengths of sequenced clones were greater than 1,000 bp, two sets of primers were designed. Genomic DNA was isolated using DNeasy Plant Mini Kit (Qiagen) as recommended, from the seed parent (S36-1-100), pollen parent (S10-2-27/28) and six segregating progeny. PCR was used to amplify gene fragments from each of the lines in a 20 μl reaction volume containing 10-50 ng genomic DNA, 1.0 U of Roche *Taq *Polymerase, 1 × reaction buffer containing 1.5 mM MgCl_2 _and supplemented with 0.2 mM dNTPs and 1.0 μM each primer. Thermocycling consisted of 5 min at 94°C; 7 cycles of 30 s at 94°C, 30 s at 65°C, and 30 s at 72°C decreasing to 58°C at 1°C per cycle, followed by 25 cycles of 30 s at 94°C, 30 s at 58°C and 30 s at 72°C, followed by 7 min at 72°C. Single PCR products were gel purified from each reaction using the Qiagen MinElute Gel Extraction Kit according to the manufacturer's specifications and were sequenced using the forward primer used in PCR amplification as above. SNPs were identified by visual inspection in Sequencher v4.6 (Gene Codes Corporation) and Pyrosequencing assays were designed using the Pyrosequencing Assay Design Software v1 (Qiagen).

### Pyrosequencing assay

SNPs which were identified by direct sequencing as being heterozygous in one or both parents were subsequently mapped in the segregating mapping population using the Pyrosequencing SNP detection platform. Forward and reverse primers, and internal sequencing primers within a few bases of the identified SNP, were designed using the Pyrosequencing Primer Design Software. One of the external primers was biotinylated and 25 μl standard PCR reactions were performed as recommended, with thermocycling consisting of 5 min at 94°C, 40 cycles of 30 s at 94°C, 30 s at 58°C and 30 s at 72°C, followed by 10 min at 72°C. Biotinylated products were immobilised onto Sepharose beads and the samples were analysed using a PSQ 96MA system together with SNP reagent kits and SNP software (Qiagen). Two mapping parents and 93 segregating progeny were scored and mapped using the methodology developed by Brennan *et al. *[15].

### Simple sequence repeat identification and analysis

SSRs were identified using the Sputnik programme and primers were designed using Primer 3 software [59]. Each 10 μl PCR reaction contained 20 ng genomic DNA, 1 μM each forward and reverse primers (one of which was labelled with 6-FAM), 0.2 mM dNTPs, 1.0 U of Roche *Taq *Polymerase, in 1 × reaction buffer containing 1.5 mM MgCl_2_. Thermocycling conditions were 5 min at 94°C, 7 cycles of 30 s at 94°C, 30 s at 65°C, and 30 s at 72°C decreasing to 58°C at 1°C per cycle, followed by 25 cycles of 30 s at 94°C, 30 s at 58°C and 30 s at 72°C, followed by 7 min at 72°C. Following PCR, fragments (along with 500 ROX-labelled size standard) were separated on an ABI Prism 3730 DNA Analyzer according to the manufacturer's instructions (Applied Biosystems). Genotypes were identified using GeneMapper v3.7 software (Applied Biosystems).

### Genetic mapping and QTL analysis

As discussed in Brennan *et al. *[15], the mapping population S36-1-100 × S10-2-27/28 contains both sibs from these parents and selfs from SCRI S36-1-100. The new markers were added to the existing linkage map using JoinMap 3 [60] to combine data from the selfs and sibs, as described [15]. QTL analysis of the budbreak data was carried out with the new map and the sibs from this cross using MapQTL 5 [61], as described in Brennan *et al. *[15]. The new markers were also tested using a more powerful mixed model analysis that combines the sib and self phenotypic data in a QTL by environment (year) analysis of the budbreak data from 2002 to 2003, as described [23].

## Authors' contributions

PH helped conceive the study, provided advice on the experimental design and molecular biology, and carried out the microarray data analysis and interpretation. JR conceived the study and coordinated the molecular work and mapping analysis. LJ performed the molecular work and helped draft the manuscript. SG collected samples and interpreted the meteorological data. JM performed all the microarray processing. CH analysed the mapping data. LC provided informatics support. RB conceived the study and coordinated the project. PH, JR and RB drafted the manuscript, which all authors read and approved.

## Supplementary Material

Additional file 1 ***Ribes *sequences with significant gene expression profiles**. Significant genes following ANOVA analysis: GenBank, GenBank accession number; dbEST, dbEST accession number; Homologue, significant blastx match; Homologue accession, public database accession number of significant match; *Arabidopsis *homologue, description of significant TAIR match; *Arabidopsis *homologue accession, TAIR accession number; Normalized, fold-change; t-test P-value, measure of significance; week 0-16, sample time point; Cluster, K-means group (see Figure 2). Click here for file

Additional file 2 **GO term enrichment within four main gene clusters**. AmiGO Term Enrichment was used to identify annotation groups significantly enriched in each of the four gene cluster sets (Figure 2) compared to the background whole microarray set. Only significantly enriched terms (see Methods) are shown. Click here for file

Additional file 3 **Quantitative RT-PCR assay details of selected genes**. GenBank & Homologue, as Supplementary Table 1; Primer1 & Primer 2, sequences of assay primers; UPL probe, sequence of probe from Universal Probe Library (Roche); Amplicon, PCR product size. Click here for file

Additional file 4 **Microarray and Quantitative RT-PCR expression profiles of selected *Ribes *genes**. Additional microarray gene expression profiles (red graphs a) & b)) and validation by Q RT-PCR (blue graphs a)). Name indicates most significant derived protein homologue, along with GenBank (GT) accession number. Scale: x-axis, time of sampling (weeks); y-axis, fold-change (log scale). Bars represent standard error. Click here for file

Additional file 5 ***Ribes *sequences selected for complete sequencing & Pyrosequencing assays**. GenBank, as Supplementary Table 1; Contig size, total length of assembled EST sequences; Primer pairs 1/2, oligonucleotide primers for Sanger sequencing; SNP type, Single Nucleotide Polymorphism alleles; Name, cross-reference code; SNP Pyrosequencing primers, oligonucleotides used to generated amplicons for SNP assay ([bioteg], biotinylated primer); Sequencing primer, used in Pyrosequencing assay; Mapped, if the sequence has been placed on the *Ribes *linkage map. Click here for file
