# Supplementary material for: Candidate genes associated with bud dormancy release in blackcurrant (Ribes nigrum L.)
Source: BMC Plant Biol. 2010 Sep 14;10:202. doi: 10.1186/1471-2229-10-202 (PMC2956551; doi:10.1186/1471-2229-10-202)
Supplement: Additional file 4 — Microarray and Quantitative RT-PCR expression profiles of selected Ribes genes. Additional microarray gene expression profiles (red graphs a) & b)) and validation by Q RT-PCR (blue graphs a)). Name indicates most significant derived protein homologue, along with GenBank (GT) accession number. Scale: x-axis, time of sampling (weeks); y-axis, fold-change (log scale). Bars represent standard error. [file 1471-2229-10-202-S4.PDF]

a)

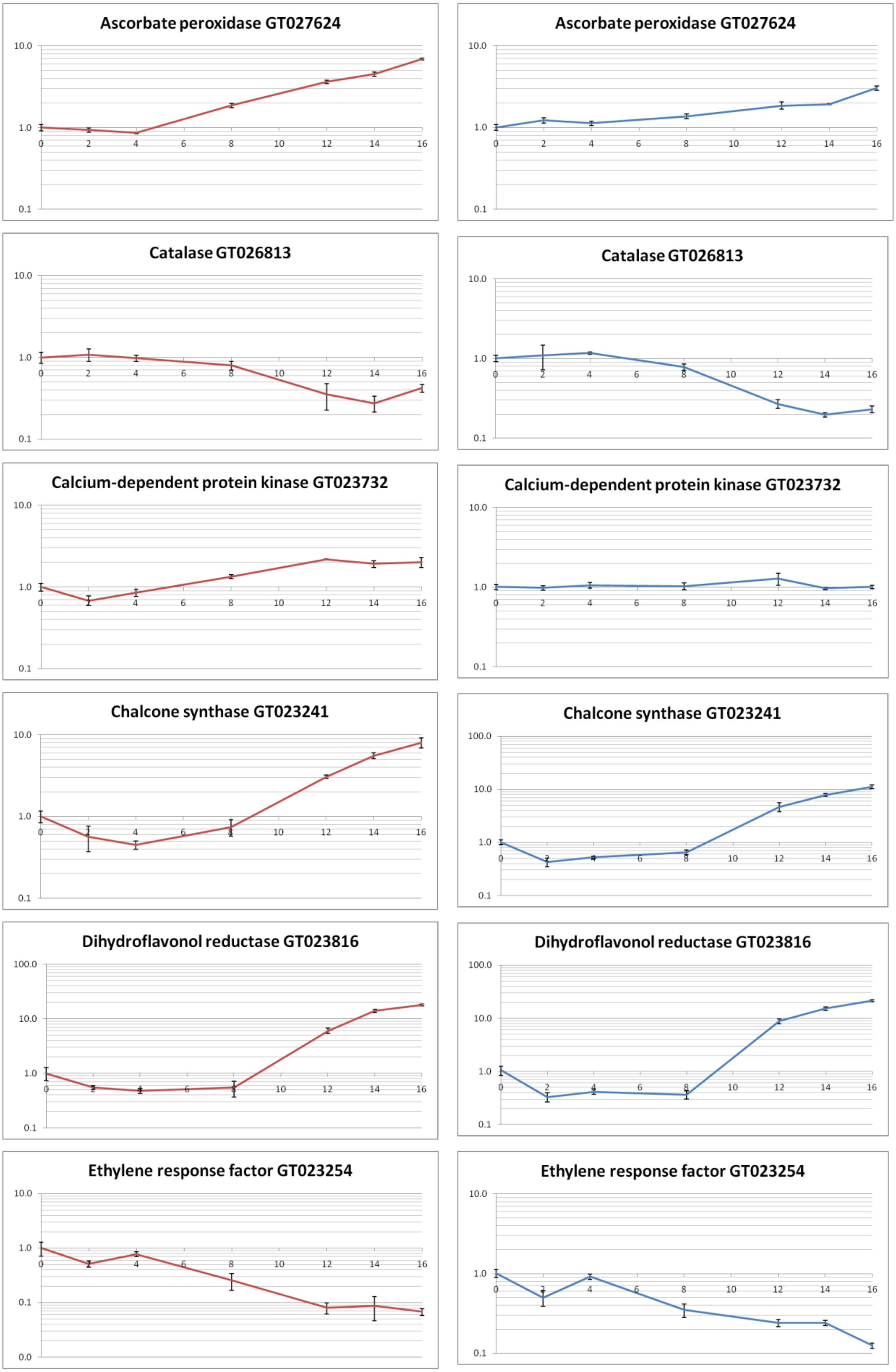
Ascorbate peroxidase GT027624
10.0
1.0
0.1
0 2 4 6 8 10 12 14 16
Ascorbate peroxidase GT027624
10.0
1.0
0.1
0 2 4 6 8 10 12 14 16
Catalase GT026813
10.0
1.0
0.1
0 2 4 6 8 10 12 14 16
Catalase GT026813
10.0
1.0
0.1
0 2 4 6 8 10 12 14 16
Calcium-dependent protein kinase GT023732
10.0
1.0
0.1
0 2 4 6 8 10 12 14 16
Calcium-dependent protein kinase GT023732
10.0
1.0
0.1
0 2 4 6 8 10 12 14 16
Chalcone synthase GT023241
10.0
1.0
0.1
0 2 4 6 8 10 12 14 16
Chalcone synthase GT023241
100.0
10.0
1.0
0.1
0 2 4 6 8 10 12 14 16
Dihydroflavonol reductase GT023816
100.0
10.0
1.0
0.1
0 2 4 6 8 10 12 14 16
Dihydroflavonol reductase GT023816
100.0
10.0
1.0
0.1
0 2 4 6 8 10 12 14 16
Ethylene response factor GT023254
10.0
1.0
0.1
0.0
0 2 4 6 8 10 12 14 16
Ethylene response factor GT023254
10.0
1.0
0.1
0 2 4 6 8 10 12 14 16

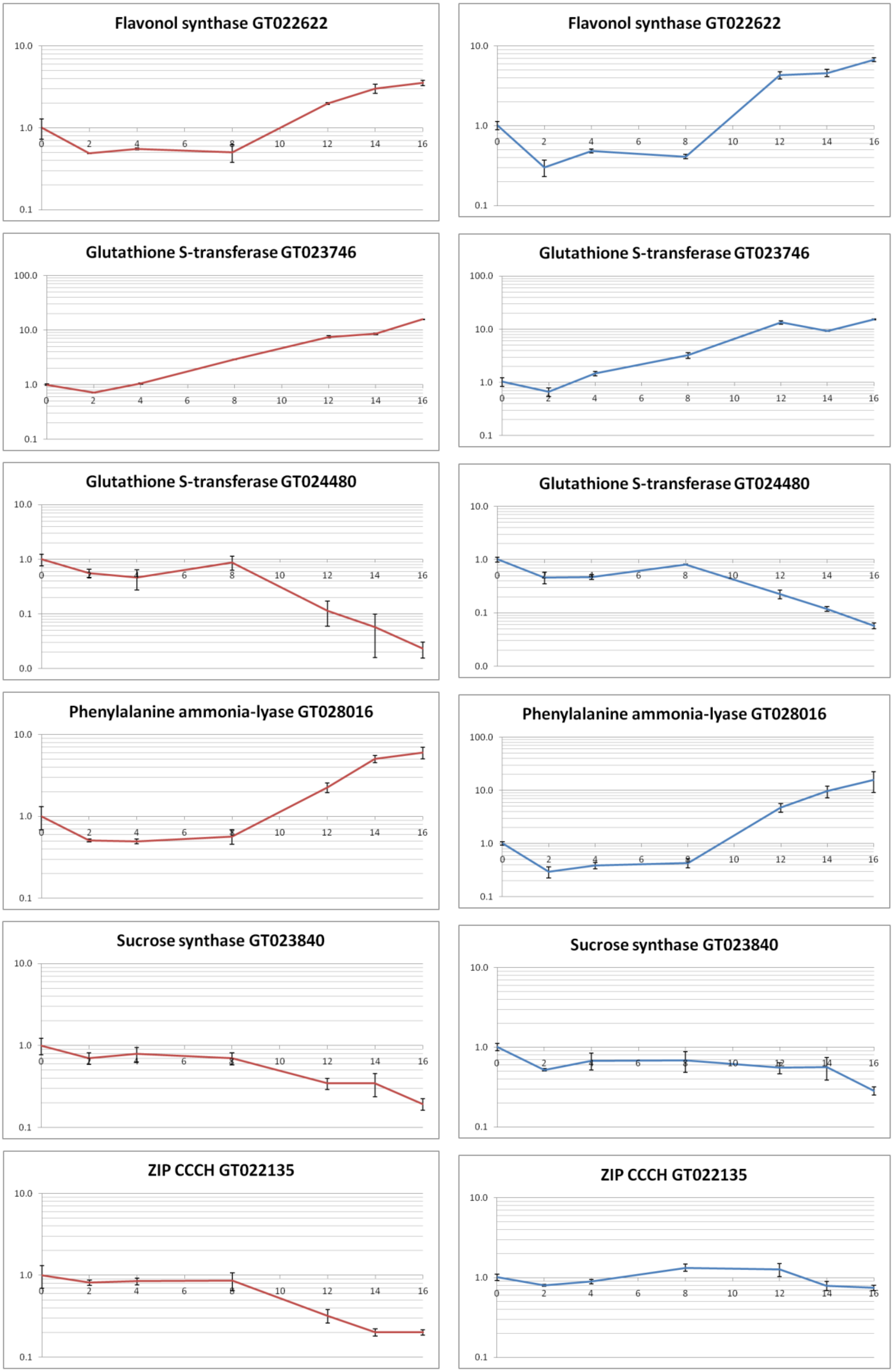
Flavonol synthase GT022622
10.0
1.0
0.1
0 2 4 6 8 10 12 14 16
Flavonol synthase GT022622
10.0
1.0
0.1
0 2 4 6 8 10 12 14 16
Glutathione S-transferase GT023746
100.0
10.0
1.0
0.1
0 2 4 6 8 10 12 14 16
Glutathione S-transferase GT023746
100.0
10.0
1.0
0.1
0 2 4 6 8 10 12 14 16
Glutathione S-transferase GT024480
10.0
1.0
0.1
0.0
0 2 4 6 8 10 12 14 16
Glutathione S-transferase GT024480
10.0
1.0
0.1
0.0
0 2 4 6 8 10 12 14 16
Phenylalanine ammonia-lyase GT028016
10.0
1.0
0.1
0 2 4 6 8 10 12 14 16
Phenylalanine ammonia-lyase GT028016
100.0
10.0
1.0
0.1
0 2 4 6 8 10 12 14 16
Sucrose synthase GT023840
10.0
1.0
0.1
0 2 4 6 8 10 12 14 16
Sucrose synthase GT023840
10.0
1.0
0.1
0 2 4 6 8 10 12 14 16
ZIP CCCH GT022135
10.0
1.0
0.1
0 2 4 6 8 10 12 14 16
ZIP CCCH GT022135
10.0
1.0
0.1
0 2 4 6 8 10 12 14 16

b)

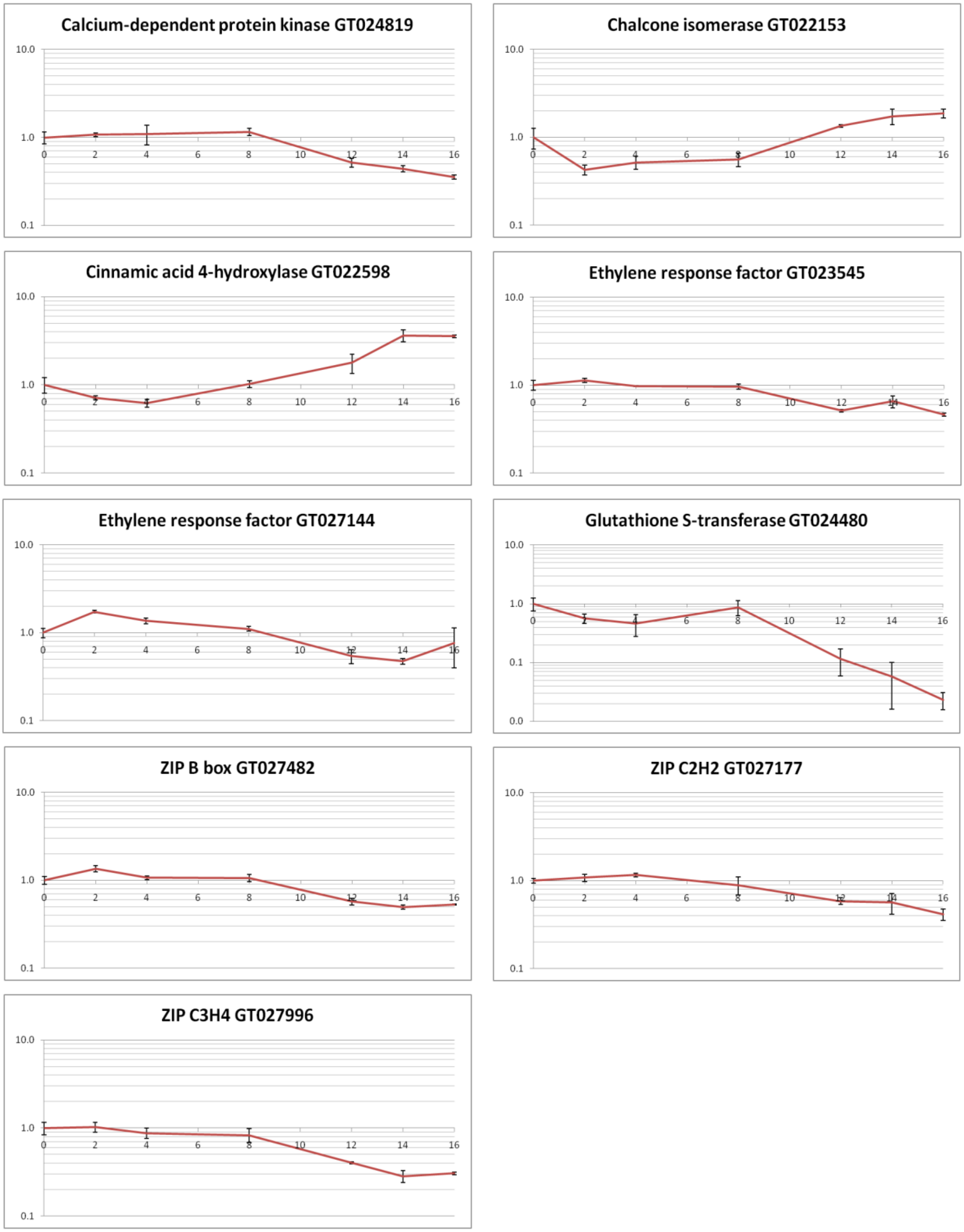
Calcium-dependent protein kinase GT024819
Chalcone isomerase GT022153
Cinnamic acid 4-hydroxylase GT022598
Ethylene response factor GT023545
Ethylene response factor GT027144
Glutathione S-transferase GT024480
ZIP B box GT027482
ZIP C2H2 GT027177
ZIP C3H4 GT027996
10.0
1.0
0.1
0.0
0
2
4
6
8
10
12
14
16
